# Chemical Pollution in Healing Spaces: The Decalogue of the Best Practices for Adequate Indoor Air Quality in Inpatient Rooms

**DOI:** 10.3390/ijerph16224388

**Published:** 2019-11-10

**Authors:** Marco Gola, Gaetano Settimo, Stefano Capolongo

**Affiliations:** 1 Architecture, Built environment and Construction engineering Dept., Politecnico di Milano, 20133 Milan, Italy; stefano.capolongo@polimi.it; 2 Environment and Health Dept., Istituto Superiore di Sanità, 00161 Rome, Italy; gaetano.settimo@iss.it

**Keywords:** decalogue, indoor air quality (IAQ), inpatient room, chemical pollution, best practices, management and design strategies

## Abstract

Indoor air quality (IAQ) is one of the main topics in which governments are focusing. In healthcare facilities, several studies have reported data analysis and case studies to improve users’ health. Nowadays, although many studies have been conducted related to the biological and physical risks, the chemical risks have been less investigated and only in some specific functional areas of the hospitals. Starting from some systematic reviews and research works, this paper aims to list the best healthy practices for an adequate IAQ in inpatient wards. In particular, the decalogue lists the strategies related to chemical pollution, starting from design and management, with a focus on (a) localization of hospitals and inpatient rooms, (b) hospital room, (c) microclimatic parameters, (d) ventilation systems, (e) materials and finishing, (f) furniture and equipment, (g) cleaning products and activities, (h) maintenance and (i) management activities, and (l) users and workers. The multidisciplinary approach emphasizes the need for interdisciplinary knowledge and skills aimed to find solutions able to protect users’ health status. The design and management decision-making, ranging from the adequate choices of construction site and hospital exposure, finishing materials, cleaning and maintenance activities, etc., which can affect the IAQ must be carried out based on scientific research and data analysis.

## 1. Introduction

### 1.1. Background

Public health is a right that must be preserved. Among the several issues that affect the quality of population health, and starting from the “One Health” approach, which is aimed at designing and improving strategies, actions, legislation, and research that involves multiple sectors for achieving better public health outcomes, healthcare facilities have a great impact in health promotion. The quality of these complex constructions is not only related to the activities provided and the multitude of daily users, but is also related to the healthiness of the hospital facility itself and the engineering plants that work 24/7 [1]. In addition, these buildings must respond to the current and future social demands, and at the same time guarantee economic and environmental sustainability.

For this reason, several professionals from different fields of interest have the duty to guarantee health promotion in the decision-making and design process of hospital settings, starting from managers, to the workers (both medical and non-medical staff), service providers, maintainers, and designers of the layouts, finishing choice, engineering plants, etc. In particular, the responsibility of hospital planners is high because their design choices can affect the performance of the hospital and its processes [2].

Nowadays, improving ‘indoor air quality’ (IAQ) is one of the goals of the UN (United Nations) Sustainable Development (refer to United Nations, Sustainable Development Goals, point 7 “Affordable and clean energy”—ensure access to affordable, reliable, sustainable and modern energy). In addition, over the last two decades, this issue in healing spaces has received growing attention. In fact, the trends in hospital design and management as well as the cleaning activities and energy efficiency strategies, have seen an increase in the use of materials and products with chemical substances; as a consequence they can be particularly dangerous for the users, if not properly selected and managed [3].

Currently, to protect worker health in the EU, some countries have set limit values for specific hard functional units that are related to physical, chemical, and biological contaminants of indoor air [4,5].

In general, as for the healthcare providers, the administrative staff, the training and teaching personnel, and the patients and visitors of hospital environments, biological hazards and related risks are mainly considered [6,7]. Furthermore, chemical contamination has become an important issue in healthcare, but is still rarely monitored and explored; for this reason, it needs to be further investigated, verified, and supervised [4]. Nowadays, several controls have begun in different countries to verify the role of chemical pollutants related to human activities, internal sources (finishing materials, furniture, etc.), HVAC (Heating, Ventilation and Air Conditioning) systems, and their maintenance, cleaning activities, energy performances, etc. [8] as well as the energy efficiency strategies that strongly affect the indoor air when they are not properly applied, or when the air changes are not controlled, especially in healthcare settings where there are several vulnerable patients.

For a better understanding of the issues that are possibly related to IAQ, it is necessary to distinguish the working areas where chemicals are used (i.e., sterilization, operating rooms, etc.), to those where the limit values of risk assessments have been already stated and adopted (occupational exposure limit values—VLEP, threshold limit value—TLV of the Scientific Committee on Occupational exposure limits—SCOEL EU or TLV^©^ of the American Conference of Governmental Industrial Hygienists—ACGIH), and those that are common in generic spaces (inpatient wards, outpatient areas, classrooms for teaching, libraries and meeting rooms, etc.) [9]. In particular, the above mentioned risk assessments can be assessed not only through the guide-values, but also through the features of the exposed variables (age, time spent, etc.), which are related to the guideline values or the references [10].

The complexity of these risk assessments requires the development of monitoring methods that consider both the specificity of indoor contaminations and the exposure levels for users [4]. Recently, the scientific literature has recorded several studies that have highlighted the differences between the various monitoring strategies used for assessing professional risks [9,11,12], and those used for indoor environments of ordinary healing spaces [13].

### 1.2. The Hospital Wards and the Inpatient Room

The configuration of the inpatient room as well as the trends of hospital design and health care are characterized by several factors that can be divided into environmental (dimensions and design features), management (related to health procedures, cleaning and maintenance activities, etc., which have to be performed), and social factors (ensuring a hospitable space for users).

Starting from the current knowledge, several impacts of environmental features on health and wellbeing in healthcare facilities can be subdivided into environmental safety, indoor air and thermo-hygrometric parameters, proper and efficient (natural or mechanical) ventilation, noise, finishing materials and furniture, viewing and experiencing nature, (natural) lighting, color, ergonomics, accessibility, wayfinding, etc. [14].

Inpatient wards are typically based on standard units of about 28–32 beds, equipped with all the necessary and main comforts. Each hospital ward has support services for medical activities and staff such as a nursing station, the head nurse office, a kitchen, storage, storage facilities, public and personal restrooms, ambulatories, and doctor offices.

The impatient ward is considered as a low–medium care environment of the hospital facility, and its users are typically vulnerable and in weak health condition.

Among the requirements to be guaranteed in inpatient wards is that it is necessary to follow the regulations regarding the finishing materials in terms of hygienic conditions. The norms define the performances that specific materials should have in order to be used in functional units.

As required by local health rules and building regulations in Italy, it is necessary to respect a minimum height limit (around 2.7 m) and an adequate ventilation and lighting ratio (around 1/8 and 1/10) [15], although the natural ventilation can be replaced by a ventilation system.

In conclusion, regarding the maintenance and the efficiency of spaces, some countries have set some limitations on cleaning products. Especially in Italy, Ministerial Decree (18/10/2016, No. 262) states the minimum environmental criteria (MEC) [16] for the sanitation activities in healthcare facilities and for the supply of detergents.

### 1.3. Scope of the Paper

Literature reviews by Gola et al. [17] and Śmiełowska et al. [18] highlighted that indoor sources of chemical pollution may include building and finishing materials and furniture, cleaning practices, use of certain chemical agents as part of medical treatments, and human occupants.

Currently, IAQ is one of the UN (United Nations) Sustainable Development Goals. In relation to multiple facets of the issue, a research group has developed a research project to monitor the activity of IAQ in inpatient wards considering the chemical influence to understand the current values and rooms’ features, maintenance activities and medical procedures, and the efficiency of the services [19,20]. Starting from the research work [17,20], the scope of this paper is to list a decalogue of design, management, and operative strategies for supporting healthcare organizations as well as hospital planners in the best performances of indoor air, especially in inpatient rooms.

## 2. Decalogue for Healthy Air Quality in the Indoor Air of Inpatient Wards

### 2.1. Localization of the Healthcare Facility and the Inpatient Ward

It is well-known that the location of a hospital is crucial to the relationship between indoor and outdoor space. Particularly, in order to obtain an adequate IAQ—the least polluted as possible—it is recommended that the concentration of pollutants in outdoor air is lower than the amount corresponding to confined environments [4,21].

Therefore, to reduce the possibility of outdoor concentrations of chemical pollutants in hospital environments, the choice of a site where such sources are minimal is relevant. However, air filtration and the location of outdoor air intakes of the ventilation system can be considered as another means by which to reduce the input of contaminants coming from outdoor air into the indoor environment [22].

When outdoor air enters a building, it imports several outdoor pollutants into the indoor environment. For this reason, the quality of indoor air is greatly influenced by the respective outdoor quality value. Sources outside the building include air pollution, emissions from heavy traffic, parking areas, or activities such as industrial processes [23,24,25].

Typically, inpatient wards are located in the upper floors of healthcare facilities, and their location is defined by the distribution of user flows. They are composed either of one-face, two-face corridors, or a double corridor, where the inpatient rooms are on the external sides. As defined by Schaefer [26]:

- the one-face corridor has a linear development and its internal distribution is characterized by inpatient rooms and a corridor. Nevertheless, it allows proper orientation of the inpatient rooms in order to obtain the best possible sunlight exposure;
- the two-face corridor is characterized, instead, by a corridor that links the services on one side with the inpatient rooms on the other. It displays some issues as far as the flux differentiation between medical and visitors is concerned. Nevertheless, it allows proper orientation of the inpatient rooms in order to obtain the best possible sunlight exposure; and
- the double corridor has a central area devoted to services and connected by two corridors to the inpatient rooms. In this case, the patient benefits from direct or indirect sunlight throughout the entire day whereas the other environmental units such as medical offices, nurse stations, kitchenette, storages, etc. constitute the central core.

In terms of IAQ, it is not compulsory to have a specific location of the rooms inside the hospital, but it is necessary to consider that:

- if the windows are closed, the location of ventilation systems and the air filtering treatments are very strategic for the air quality;
- if the windows can be opened, then it is necessary to consider the vulnerable users: (a) the contribution of the ventilation system; (b) the size of the windows and their openings; (c) the neighborhood where the building is located (traffic area, factories, etc.) [27], and in particular the proximity of the front; and (d) the floor where it is located and the possible influence of wind.

The location of the room, with respect to the building typology and the organization of the project area, can have different solar exposures. From the technological and energetic points of view, the optimal location of the ward should be on the north–south axis, so that the rooms would have a western and an eastern exposure. The contribution of solar exposure, if not properly controlled in terms of technology (i.e., shielding system) and engineering plants (i.e., HVAC), is able to cause overheating indoors, and a greater emission of pollutants from finishing materials within the environment.

As the scientific literature and several case studies show concerning the pollution of the neighborhood and the solar exposure, strategies in ventilation systems with high performances and filtering systems, and technological strategies for reducing solar intake can be applied [17,22,23,28,29,30].

Although, the façades of healthcare facilities are commonly modular and regular, and typically equal among the various fronts, in relation to the solar exposure, designers can apply different technological strategies for reducing solar intake: (a) openings of different sizes (respecting the minimum lighting requirements); (b) external shields (brise soleil, mechanical systems, external projections, etc.); (c) volumes and/or recesses on the façade; (d) windows with low emissive, selective, thermal glasses, etc.; (e) shielding systems integrated into the windows, despite the fact that they can cause overheating inside the room, difficulties for maintenance, etc.; and (f) internal shielding systems, even though they are not recommended for hygiene aspects, user safety, and overheating of the internal environment.

### 2.2. Hospital Room

Hospital rooms require attention because they should meet the needs of patients, healthcare staff, and visitors. Well-designed hospital rooms speed up healing (as experiences of evidence based design have demonstrated) [31,32] and, in addition, decrease the rate of errors in medical process [33]. A user-centered design can benefit everyone: for the patients, the room is a healing place, a bedroom and a living room; for the healthcare staff, it is a workspace where several medical activities and procedures take place; and for visitors, it is a living room and a bedroom. As a consequence, multi-functionality should be the keyword in a place where the basic requirements are multiple and the space is restricted:

- the first area is the Service Zone. It includes the bathroom and a space specifically dedicated to nursing activities. The bathroom should be designed according to (disabled) accessibility regulations. In addition, the staff area entrance should be restricted. This first zone has a lower false ceiling in order to save space for installations and ventilation systems;
- in the Patient core, all attention is focused on the patient. Behind the bed, there are medical gases, reading lights, and electrical plugs. There is typically, at least, a wardrobe for personal items; in addition, in new and international hospitals, there is an interactive screen and a small screen to let the doctor access all information and data;
- the third area (optional) is called the Family Zone. Usually, the relationship between visitors and patients is solved by placing an armchair in a corner. In this case, space and furniture are the only elements that allow visitors to be hosted.

There are no specific dimensions for double and single rooms, but generally the average size of an inpatient room is around 25 m^2^, bathroom included. Escombe et al. stated that isolation rooms should have a volume equal to 31 m^3^ [34], but this dimension is strongly affected by several variables defined by ventilation systems, human density, solar exposure, window openings, etc.

Door motion can influence the IAQ because it affects the air volume exchanged between two spaces, and increases when the movements of the users are combined [35,36]. Kalliomäki et al., in their analysis on isolation rooms, demonstrated that the exchange volume was significantly lower with sliding doors than with hinged ones, although it is well-known that the sliding solution has worse hygienic conditions [37].

In addition, as Sehulster et al. demonstrated, the leakage area in a room is set around 0.03 m^2^. This is suggested to guarantee an undercut of about 1–1.5 cm; undercutting minimizes resuspension due to the door scraping the floor [38]. The minimum pressure difference between rooms and corridors, rooms and toilets, is around 2.5–6 Pa [38]. In the case of sealing doors, ventilation grilles should be installed.

For lighting requirements, the windows are compulsory in inpatient rooms, but at the same time, it is not mandatory that they can be opened. From an economic and energetic point of view, as several hotel facilities have already designed, the windows are closed to guarantee high efficiency in the facility [1,4,22]. If the windows cannot be opened, then it is extremely necessary to have an efficient HVAC that allows for regular air exchange and dilutes the pollutant concentrations.

Starting from the existing state of the art of different glass types to reduce overheating, and considering the studies about the influence of the color of glass on users’ wellbeing, it is suggested that these aspects in design strategies are considered for the best efficiency of the room [30].

Starting from the previous considerations, as several authors have suggested, to create a well-designed environment and, at the same time, have adequate IAQ, it would be necessary, as Gola suggests [39]: (a) to use and supply specific durable materials and surfaces that facilitate cleaning and maintenance activities with high performances [40]; (b) to design an anteroom as a means by which to reduce the concentration of pollutants in the air from the air containment and migration to protect the adjacent corridors through an excess of air flow in or out of the room [37]; (c) to maintain a specific air flow rate between the inlet and discharge [22,29]; and (d) to ensure that the flow of air from one space to another takes place through the slots in the walls, ceilings, floors and around the doors.

Therefore, for an adequate IAQ in these rooms, it is necessary to pre-assess the possible factors that influence the performances and impacts related to selected finishing materials and furniture, human activities, typology and frequency of cleaning activities, microclimatic parameters, ventilation system, and the volume of the room.

### 2.3. Microclimatic Parameters

The systematic review by Gola et al. [17] highlighted the role of microclimatic factors such as temperature and humidity. In fact, hospital air quality indices include temperature and humidity, levels of CO_2_, total volatile organic compounds (TVOCs), particulate matter (PM_10_ and PM_2.5_), bacteria, etc. Both temperature and relative humidity are recommended indices to reflect the efficiency of ventilation systems in healthcare facilities; however, they do not provide information on how separate parameters such as air change rate and increase of surface area by the furniture in environmental units may affect the deposition of particles and, therefore, the relationship between the distribution of aerosol particles in the room [41,42].

Microclimatic parameters (temperature, relative humidity, and air velocity) have a great influence on indoor air in healing environments. For example, several institutions state guidelines and reference values for adequate comfort and IAQ in inpatient rooms such as temperature during the year should be around 21–24 °C, the relative humidity should be around 40–60%, and the air velocity should be between 0.05 m/s and 0.20 m/s for heating, and between 0.05 m/s and 0.25 m/s for cooling, etc.

As shown in the scientific literature, the increase in microclimatic parameters can affect the performance of finishing materials and furniture as well as the chemical emissions of cleaning products.

An adequate control of microclimatic parameters supported by the presence of window openings and efficient ventilation system can guarantee good performances in IAQ and user comfort.

### 2.4. Ventilation System

In inpatient wards, the influence of medical activities, user health status, high temperature for patient wellbeing, etc. can affect indoor air performance. It is therefore necessary to have an adequate ventilation system to control indoor air pollutants by reducing and removing them by dilution, filtration, and air purification of IAQ.

HVAC ventilation systems should be designed specifically for healthcare environments and not for other confined environments such as offices, living areas, etc. As Pati et al. [43] observed in an analysis related to exam rooms, ventilation systems should be designed specifically to carry out the activities and should be managed (and well-filtered) to guarantee better performance during their operation.

As seen in the scientific literature, insufficient ventilation and filtration influence poor IAQ at the entrance of external contaminants in the building, the recirculation of internal pollutants, and dirt build-up in air handling systems.

The ventilation within a healthcare facility is typically forced, but in several existing case studies, natural ventilation has been guaranteed by window openings and window frames. Recent case studies, however, have been to seal them for a reduction in energy dispersion and expense, although these strategies have caused a drastic reduction in air-flow rates and thus an increase in the concentrations of pollutants [29]. Therefore, perfect sealing fixtures cause high concentrations of pollutants in environments without air exchange.

To ensure an efficient ventilation system that is able to reduce the concentration of pollutants in hospital rooms: (1) the amount of outdoor air should be appropriate for each environment in relation to the number of occupants and the activities carried out; (2) a minimum filtration efficiency of high-medium value for the first filter and high level value for the second filter must be met, and an air exchange rate of about 6 v/s, as ASHRAE (American Society of Heating, Refrigerating and Air-Conditioning Engineers) suggests [44]; (3) ventilation must be well distributed in the breathing zone of users; and (4) if possible, polluted air should be extracted through extraction systems.

Regarding ventilation rates, two distinct ventilation rates are typically required, as mentioned by ASHRAE [44]: (1) Per person, diluting pollution sources associated with users’ activities should be considered proportional to the number of occupants; and (2) per unit of surface, diluting the emission of contaminants of building materials, furniture, and other sources not associated with the number of occupants.

In conclusion, among the maintenance strategies for ventilation, at the end of the construction of a new building, the emissions of contaminants from building materials and finishing are generally higher [45]. To mitigate this, it is useful that the conditioning system operates for a period at a rate greater than the normal one in order to purify the air from the contaminants before human occupation and during initial occupation [46]. In any case, the engineering plants must also be designed to ensure adequate ventilation even during cleaning and any other polluting activities [4].

### 2.5. Materials and Finishing

In order to minimize the sources of IAQ, it is important to identify and select materials that respond to medical and hygiene requirements as well as consider the fact that the environmental conditions of the room can influence the release of pollutants (i.e., solar exposure, high-temperature, or humidity can increase material emissions, etc.).

For the control of emissions from construction materials, it is necessary to adopt preventive practices in order to guarantee protection against mechanical damage or chemical pollution during transport, on-site storage, and the installation of materials used. It is necessary to ensure adequate ventilation, no direct sunlight, and moderate temperatures (antifreeze protection). Particularly, porous materials must be protected from moisture and dust as well as gases and vapors during their storage.

Assessing impacts due to contaminant emissions, materials should be considered as part of systems. The emissions of a complex system can be considerably different from those of individual components. In addition, environmental conditions can influence the release of pollutants (i.e., high-temperature materials or high humidity are able to increase emissions [47]).

In any case, to achieve the healthiest possible environments, the materials that should been chosen should have: (a) a low emission of VOCs (volatile organic compounds) and formaldehyde that has been tested and certified by qualified institutes according to international standards; (b) to be as least porous as possible to facilitate cleaning and thus reduce the effects of detergents and disinfectants; (c) little maintenance and less application of chemicals required for surface renewal. In general, materials must comply with European certifications such as Deutsches Institut für Bautechnik (DIBt), Ausschuss zur gesundheitlichen Bewertung von Bauproukten (AgBB), French Agency for the Protection of Environment and Occupational Therapies (AFSSET), Gemeinschaft Emissionskontrollierte Verlegewerkstoffe (GEV), etc.

As the scientific literature shows, to achieve healthier environments, it is necessary to have an adequate selection of materials with low VOCs emissions, and materials that have as low a porosity as possible to facilitate cleaning activities and ease of maintenance [48].

Designers can avoid introducing potential sources of air pollution in indoor environments by carefully selecting materials that have minimal health risks [49]. Therefore, it is necessary to select products based on the following factors: data on chemical composition and emissivity; safety and/or technical data sheet; cleaning and maintenance information; and instructions for storage, transportation and installation [45].

Highly absorbent materials should be installed after the completion of construction activities that release high VOC levels (e.g., paints, adhesives, and sealants, etc.). If the installation phases are not carefully monitored, these materials can act as contaminant tanks that release long-term re-emissions into the indoor air.

In order to reduce the emissions accumulated during installation, the environment must be equipped with additional ventilation for a minimum of 72 h after installation (i.e., PVC (polyvinyl chloride), unlike linoleum, requires maintenance during its lifecycle, since waxing is not necessary to be equipped with surface treatments).

Placing new materials in a well-ventilated area and in a clean space before installation can be an effective means in which to reduce high emissions. Despite the efforts to limit materials with high emissions of pollutants, there are no non-toxic or ideal materials, and the use of certain materials and products with moderate emissions may be necessary for the specific hygiene requirements of hospital facilities [20].

In general, finishing materials must comply with international certifications. During and after their installation, they need adequate (natural) ventilation to dilute their emissions, which may be for several days. The finishing materials used for ceilings, walls, and floors, due to their large surfaces, can have a direct impact on IAQ through their pollutant emissions. The use of zero or low release VOCs, formaldehyde, or other chemical emissions on the ceilings and walls would reduce chemical risks, thus improving the quality of indoor air.

When considering the use of a resilient material, the US EPA (United States Environmental Protection Agency) recommends testing for VOC emissions through adequate programs; it should be easily cleaned and maintained with low-quality detergents and finishes; installed with low VOC adhesives in order to minimize the health hazards due to air pollution for the installers and occupants; and is installed using the minimum required amount of adhesive to meet the product performance requirements.

### 2.6. Furniture and Equipment

In order to reduce the sources of indoor air pollution, it is important to identify and select furniture and equipment that respond to the hygiene requirements and to the medical activities to be carried out.

As several experts have suggested, an adequate selection of furniture with low VOCs emissions is necessary and should have the lowest porosity possible to facilitate cleaning activities and easy maintenance. Furniture should be placed in indoor environments after the site construction has been completed [50].

Currently, a commonly used material for easy cleaning and maintenance as well as durability and safety features is PVC. However, given the non-natural composition, this material releases a wide range of VOCs including plasticizers and solvent residues. The use of zero or low release VOCs, formaldehyde, or other chemical emissions on the ceilings and walls would reduce chemical risks and improve the quality of the indoor air.

Nowadays in inpatient rooms, in many hospitals, PVC based furniture is widespread because it is less expensive than other materials, is easy to clean, and requires less maintenance. However, it is not a material that guarantees good IAQ due to the wide range of VOCs released during its lifecycle [51].

Furniture must comply with international certifications, during and after their installation, and they need adequate ventilation to dilute their emissions for several days (at least 72 h). In addition, in order to avoid the emission of contaminants due to deterioration, finishing materials and furnishings must be periodically checked and maintained. It is crucial to ensure that hospital equipment and furnishings are placed in positions where they do not interfere with airflows.

Workers must ensure that they do not release fibers and particles during proper installation, which must be carried out according to the instructions of the manufacturer.

In conclusion, medical equipment can be a potential source of phthalate emissions into the air (commonly used as plasticizers and polymer materials such as plastic infusion bags, blood bags, plastic film, injectors, and rubber tubing, etc.), especially if they are located near the breathing zone of patients when they are in use [18,51]. Managers should select materials with low VOCs emissions.

### 2.7. Cleaning Activities and Products

A clean indoor environment is considered as an essential requirement for an adequate IAQ. However, an incorrect choice of detergents and/or application practices may cause adverse effects on air performances. The simplest and most effective method to reduce the impact on cleaning products is to reduce their necessity. However, due to the high level of hygiene required in the hospital, it is difficult to reduce the use of cleaning agents. Therefore, the careful selection of minimal emission cleaning products is recommended [3]; in general, wet surface cleaning with respect to the spray method generates fewer pollutants in the IAQ [52].

As Bello et al. demonstrated, proper ventilation must be ensured during and immediately after cleaning. Several studies have suggested opening windows for 10–15 min in the room (or in the corridor), in the absence of patients [53].

The current scenario for the optimal performances of detergents for cleaning (and disinfection of surfaces) are:

- floors: neutral detergents, weakly alkaline (chlorine-derivatives, quaternary ammonium salts, phenol, etc.);
- furniture: neutral detergents for dusting (quaternary ammonium salts, phenol, alcohols, etc.);
- windows: neutral detergents (alcohols, phenol, etc.);
- bathrooms: detergents, weakly acid (chlorine-derivatives, quaternary ammonium salts, phenol, etc.) [40].

In this scenario, a strategic aspect related to the design phase is the selection of finishing materials and furniture with surfaces that are easy to clean without the use of high impact chemical agents [54,55].

Cleaning activity should be defined through specific protocols. The protocol should list the product safety data sheets, correct preparation of the solution, and proper use of the cleaning agents must be considered, and therefore application methods, contact times, and incompatible materials [19].

Proper ventilation must be ensured during cleaning and immediately after it. Studies by Bello et al. have suggested opening the window for 10–15 min. [53], if possible (the corridor windows must also be opened). In contrast, simulations by Bello et al. demonstrated that concentrations of pollutants steadily increased by time during task performance, and reached their peak at the end of the cleaning period; concentrations after the tasks declined exponentially to background concentrations [53]. The time it took to reach the previous level was about 20 min later.

In conclusion, proper cleaning staff should be trained to understand the responsibilities and health risks to themselves and to the users of healing spaces [56].

### 2.8. Maintenance Activities

It is fundamental that hospitals, in addition to being well-designed and built to ensure adequate IAQ, must be regularly maintained to ensure adequate performance of the healthcare facility. As the scientific community highlights, a regular and constant maintenance and management program can facilitate the coordination of the necessary activities to be carried out [57].

Ordinary activities are the combination of all technical and administrative actions including monitoring that is aimed at maintaining the facility in the appropriate conditions to perform the requested functions [58]. Depending on the amount of work, there are several activities that can be carried out with the presence of the user in the room, or with a temporary or prolonged suspension of the inpatient room.

Among ordinary activities, regular monitoring of ventilation systems should provide access to all components for inspection, repair, and cleaning. In addition, ensuring that the materials have durable surfaces will reduce the maintenance needs and impacts on replacement or finish related to the IAQ [58].

Extraordinary activities include the interventions for the adaptation to new regulations, extensions, and/or modifications; restructuring existing works due to (new) user requirements; and new ventilation systems or partial transformation of existing ones. They can also relate to structural changes of the layout of the room and/or the installation of devices, equipment substitutions, etc. These activities may require the suspension of medical activities, mainly in the functional unit, and sometimes of the single inpatient rooms [59].

In general, extraordinary cleaning is required, especially for dust and products adopted during these interventions. It is possible to use different products from those used for ordinary and daily cleaning activities, if they are appropriate and adequate for the materials [4].

One of the main causes of IAQ problems is premature occupation. Buildings are often occupied before the end of the works (furnishings or ventilation systems, etc.). It is essential to ensure that the ventilation and thermal control systems are active before the initial occupation and that they operate for a period of time at a ventilation rate greater than the normal rate in order to eliminate contaminants from the indoor settings, finishing, and furnishings since their emissive profile is characterized by maximum peaks in the period immediately after installation [46].

In conclusion, the actions that should be verified are, as Gola suggests: periodic inspections in healing environments and checking the quality and efficiency of spaces; identification of potential air pollution by maintenance activities; analysis of datasheet of cleaning products; and the use of construction materials, furnishings, and medical equipment with low VOC emissions [39].

### 2.9. Management Activities

It is fundamental that a hospital, as well as being well-designed and built to ensure adequate IAQ, must be well managed, to avoid that the strategies and design solutions—necessary to have healthy environments—are thwarted, and to promote health. The success of IAQ control is strongly related to the joint efforts of the engineering, healthcare, administrative, and support staff [60].

In general, an adequate IAQ management program can facilitate the coordination of the necessary activities to identify, correct, and prevent IAQ issues and overcome critical factors with design and management strategies [4]. In addition, the implementation of a management program can also raise awareness in all staff to achieve good hospital IAQ. As provided by Leung and Chan, their seven-step IAQ management program was designed for implementation in a healthcare facility [60].

Regular monitoring activities permit the healthcare organization to know how the hospital is performing. Therefore, it is suggested that comprehensive IAQ assessments with detailed measurements are conducted and that the measurements are analyzed to identify appropriate mitigation measures and strategies [61]. Active and passive samplings can be applied to understand the values of indoor air pollution in inpatient wards.

In addition, air sampling and the warranty of adequate values can guarantee that hospital managers, in the case of discomfort and complaints from workers and patients, have the necessary data to certify the correct provision of medical activities and the quality of healing environments. A qualitative analysis on the quality of the inpatient rooms can be conducted through questionnaires and surveys to collect information from hospital staff and users, especially in relation to indoor environmental quality and indoor air (odors, microclimatic conditions, solar exposure, etc.) [62].

As Leung and Chan suggest, the IAQ manager in healthcare facilities, who is responsible for the overall development and implementation of the IAQ management program, can be strategic for the definition of strategies for health promotion [60]. The manager should: (a) set up an IAQ management team comprising of several representatives from different departments in order to support the manager for strategic actions and interventions; (b) conduct periodic walk-through inspections in healing environments and verify the quality and efficiency of spaces; (c) be aware of legislative requirements, standards, and guidelines and train all hospital staff to ensure that they are well-aware of their activities and the medical procedures carried out; (d) identify the potential air pollution that could be caused by healthcare procedures and equipment; and (e) verify and require specifications, if necessary, for cleaning products, construction materials, furnishings, and medical equipment for low emissions of chemical pollutants.

### 2.10. Users in Inpatient Ward: The Role of Patients, Visitors and Sanitary and Non-Sanitary Staff

It is suggested that the adequate behavior by all users could guarantee the best performances of indoor air, although the impact of the users’ presence in the air quality of inpatient rooms is not as high when compared to other factors. As several authors have demonstrated [63,64,65], the health status and metabolic processes of patients can cause air pollution in breathing zones.

In general, all users must comply with a behavioral code, appropriate to healthcare settings, and not endanger the health of others [66]. In fact, it is evident and still common in several Italian hospitals that there is smoking inside the patient’s room; this can cause poor air quality for the users.

In general, in inpatient wards, during a day, there are several users: from hospital staff (medical one, residents, cleaners and maintainers) to patients and relatives. Physicians and nursing staff carry out medical activities and therapies amongst the equipment’s positioning (plastic infusion bags, blood bags, plastic film, injectors and rubber tubing, etc.), which could affect the quality of the user’s air in the breathing zone [18]. In addition, in the case of environmental inconveniences, they are responsible for the patients’ wellbeing [67].

Additionally, visitors can influence the air performance: in fact, their health status, possible gifts, and personal items can affect the deterioration of air quality, in addition to the number of people (human density) in the room. Furthermore, their presence in the room causes the transport of some pollutants (especially biological ones) from the outdoors [28].

Room cleaners should respond to the protocols for the quantity of products for cleaning activities, but sometimes some irregularities can be made [53]. It is therefore necessary that cleaners are trained on the risks that products can cause and to reduce all possible mistakes.

Maintenance technicians aim to verify the state of healthcare environments and their implementation. The activities carried out are related to punctual interventions in the room (furniture or finishing materials replacement, etc.) or the entire inpatient ward (cleaning of ventilation systems, etc.), therefore their responsibility is related to different scales: from the environmental unit to the functional unit or hospital building.

Therefore, strategic choices with the aim of promoting health in the work and living environments for users, both in procedures (medical, maintenance, cleaning, etc.) to be implemented, and in purchase of supplies, furniture and finishing should be applied.

## 3. Conclusions

It is efficient that IAQ is a very broad topic because any variable can affect the performances of air in indoor environments. Design and management strategies, which may be adequate in relation to different procedures and operative management methods, are able to decrease or increase the quality performances of the room and the users’ comfort.

The multi-disciplinary approach demonstrates the need of interdisciplinary knowledges and skills aimed to find solutions that are able to protect the health status of users. It is clear that the design and management decision-making depends on the adequate choices of construction site and hospital exposure, finishing materials and furniture, cleaning and maintenance activities, etc., that can affect the IAQ, and has to be carried out on the basis of scientific research and data (evidence based design) [2,32]. It is further necessary that the decision-making team should be composed of different professionals in order to guarantee a multidisciplinary and synergic design project in its various aspects.

The hospital system must be rethought and reconsidered by paying attention to pollutant emissions in all aspect, and is equipped with buildings that from the design phase to the realization and operation stages are able to maintain a safe environment. In this regard, it is worth underlining that it is not enough to define design solutions and engineering plants for emission control, but it is essential to pay attention to all the factors and procedures that are also related to user behavior [68]. It is crucial that everything starts from the design phase, by defining the aims and scope as well as the objectives to be achieved and make the most appropriate technical choices in which to attain them.

The decalogue lists the strategies for health promotion in hospital wards from the chemical pollution point of view. The best practices apply to project managers, designers, technicians, and all other users who can actively contribute to the reduction of health risks, highlighting the importance of hygienic aspects and user behavior [69]. As the analysis of the case studies showed, typical studies related to IAQ—especially related to chemical pollution—have rarely considered the potentialities of design strategies when considering the management sphere.

Therefore, programming (a), control (b), and design strategies (c) can highly affect and improve the air quality, and thus only a professional and multidisciplinary approach can guarantee good working performance [19].
